# Editorial for Special Issue: Advanced Technologies for Developing the State-of-the-Art Nanomedicines

**DOI:** 10.3390/pharmaceutics15071954

**Published:** 2023-07-15

**Authors:** Yi Hu, Jun Chen

**Affiliations:** 1 CAS Key Laboratory for Biomedical Effects of Nanomaterials and Nanosafety, Multi-Disciplinary Research Division, Institute of High Energy Physics and University of Chinese Academy of Sciences (UCAS), Chinese Academy of Sciences (CAS), Beijing 100049, China; 2 State Key Laboratory of Complex Severe and Rare Diseases, Peking Union Medical College Hospital, Beijing 100730, China

This Special Issue aims to introduce advanced technologies that promote the development of nanomedicines. In recent decades, nanotechnology has been widely applied in various areas of modern medicines, and has provided significant help in developing safe, effective, and precise drug therapies. The Special Issue collects 10 articles that explore different advanced technologies for developing state-of-the-art nanomedicines, including nucleic acid therapy, myocardial infarction, and cancer treatments. The aim of this issue is to provide a review of recent developments and encourage continued progress in this field.

The COVID-19 pandemic has sparked a huge increase in research into nucleic acid therapy, which has garnered significant attention as a potential treatment and preventive option. Nucleic acid therapy can achieve long-lasting and curative effects through gene enhancement, inhibition, editing, and other methods. However, to be effective, it requires delivery systems, since naked nucleic acids cannot enter cells effectively. This Special Issue includes a review article on nucleic acid delivery, which highlights cationic polymers as a promising delivery system due to the ease of synthesis, modification, and structural control [1]. In addition, because of the unique advantage of RNA in nucleic acid therapy, two articles on RNA delivery are also included. One article addresses the challenge of treating chronic hepatitis B, a major cause of liver diseases such as hepatocellular carcinoma (HCC). The authors show that co-delivering siRNA targeting HBV X protein (HBx) and a plasmid encoding interleukin-12 (pIL-12) can effectively inhibit virus replication, reactivate the immune system, and slow down the development of HCC [2]. This work holds great promise for improving treatment options for patients with chronic hepatitis B. Another article uses all-atom MD simulations to investigate the thermal stability of three-way junction-packaged RNA (3WJ-pRNA). The authors discover that Mg^2+^ ions can regulate the thermal stability of 3WJ-pRNA, which could be valuable in developing controllable RNA nanogranule drug delivery platforms [3]. These findings are critical for designing efficient and effective RNA delivery systems.

Small-molecule medicines have been widely used in various diseases. The nanoformulation of these medicines can fundamentally alter their in vivo bioavailability. In the treatment of myocardial infarction, nanocarriers have shown potential advantage. A review article in this issue summarizes important principles and developments in the field, focusing on the nanocarriers with ligand-based or cell mimicry-based targeting and discussing the current limitations and future directions [4]. In addition, this issue includes three papers related to cancer treatments, where nanocarriers have played an essential role. These include (1) a promising nanobiophotonic theranostic (ICG-MB) for cancer phototherapy, which is simple and biocompatible [5]. When stimulated by near-infrared laser irradiation, it exhibits enhanced photothermal conversion and singlet oxygen generation to ablate cancer cells. This cancer phototherapy candidate is self-assembled from two FDA-approved dyes without additional additives. (2) A high drug-loaded nanocarrier crystalline micelle is produced using a diblock copolymer, poly(oligo ethyleneglycol) methacrylate-b-poly(styrene-co-4-formylphenyl methacrylate) (PPP) as a stabilizer. The micelle exhibits excellent colloidal stability, controllable drug release, and prolonged circulation properties. It can induce high accumulation in tumor tissues even after repeated administration, achieving continuous antitumor efficacy [6]. (3) The modulation of M1 extracellular vesicles (MM1-EVs) loaded with doxorubicin enhances the antitumor effect of chemotherapy in a metastatic cancer mouse model [7]. MM1-EVs reduce the size and metastasis of primary tumors, decrease the expression of M2-TAMs, and enhance tumor apoptosis.

The physio-chemo properties of nanocarriers are crucial factors affecting the efficacy of drug delivery, and optimizing their properties can further enhance their prospects in the medical fields. Two research articles in this Special Issue focus on this concept. The first article seeks the optimal properties of nanoparticles (NPs) in mediating improved in vivo responses. The authors evaluated NPs prepared from five materials of three sizes and three concentrations in a cell barrier model. They found that ZnO NPs cause significant alterations to cell viability across all three cell lines tested, while NPs with a physiological-based zeta potential of −12 mV result in good cell barrier penetration without considerable changes in cell viability [8]. Another article describes the synthesis of aliphatic polyanhydrides with various hydrophobic segments, controllable molecular weights, low polydispersity, and potential for use as drug carriers. The authors synthesized a series of polyanhydrides using suberic, azelaic, sebacic, and dodecanedioic acids and achieved reduced polydispersity [9]. The molecular weights of the synthesized polyanhydrides are highly controllable and depend on the degree of activation of the dicarboxylic acid monomers, i.e., the amount of acetic anhydride used during synthesis. Furthermore, unlike traditional methods of optimizing material properties, choosing the appropriate stereoisomer can significantly impact drug efficacy and adverse reactions. Therefore, this Special Issue also includes a review article which highlights the influence of nanoparticle chirality on the interactions with biological systems and introduces the chiral materials used in nanomaterials [10].

In conclusion, this Special Issue highlights the significant progress and promising outlook for nanomedicines in various medical fields. The articles cover several topics, including nucleic acid therapy, myocardial infarction, cancer treatment, and the optimization of nanocarrier properties. These studies provide valuable insights into the development of efficient and effective medical treatments using nanotechnology. By exploring new approaches and avenues for developing novel nanoparticle-based therapies, researchers have demonstrated these materials’ vast and exciting potentials in modern medicines. With insights from these articles, we can expect continued advancements and breakthroughs in the development of efficient and effective medical treatments using nanotechnology in the future.
